# Metabolic profiling reveals altered tryptophan metabolism in patients with kawasaki disease

**DOI:** 10.3389/fmolb.2023.1180537

**Published:** 2023-05-04

**Authors:** Xue Fan, Ke Li, Xin Guo, Shengyou Liao, Qi Zhang, Yangkai Xu, Hongtu Cui, Lemin Zheng, Mingguo Xu

**Affiliations:** ^1^ Department of Pediatrics, The Third People’s Hospital of Longgang District Shenzhen, Shenzhen, China; ^2^ Advanced Innovation Center for Human Brain Protection, China National Clinical Research Center for Neurological Diseases, Beijing Tiantan Hospital, Capital Medical University, Beijing, China; ^3^ Department of Clinical Medical Research Center, Guangdong Provincial Engineering Research Center of Autoimmune Disease Precision Medicine, The Second Clinical Medical College, Jinan University (Shenzhen People’s Hospital), Shenzhen, China; ^4^ Key Laboratory of Molecular Cardiovascular Sciences of Ministry of Education, Health Science Center, School of Basic Medical Sciences, The Institute of Cardiovascular Sciences and Institute of Systems Biomedicine, Peking University, Beijing, China

**Keywords:** kawasaki disease, tryptophan metabolism, coronary arteritis, metabolomics, biomarker

## Abstract

Kawasaki disease (KD) is a childhood vasculitis disease that is difficult to diagnose, and there is an urgent need for the identification of accurate and specific biomarkers. Here, we aimed to investigate metabolic alterations in patients with KD to determine novel diagnostic and prognostic biomarkers for KD. To this end, we performed untargeted metabolomics and found that several metabolic pathways were significantly enriched, including amino acid, lipid, and tryptophan metabolism, the latter of which we focused on particularly. Tryptophan-targeted metabolomics was conducted to explore the role of tryptophan metabolism in KD. The results showed that Trp and indole acetic acid (IAA) levels markedly decreased, and that l-kynurenine (Kyn) and kynurenic acid (Kyna) levels were considerably higher in patients with KD than in healthy controls. Changes in Trp, IAA, Kyn, and Kyna levels in a KD coronary arteritis mouse model were consistent with those in patients with KD. We further analyzed public single-cell RNA sequencing data of patients with KD and revealed that their peripheral blood mononuclear cells showed Aryl hydrocarbon receptor expression that was remarkably higher than that of healthy children. These results suggest that the Trp metabolic pathway is significantly altered in KD and that metabolic indicators may serve as novel diagnostic and therapeutic biomarkers for KD.

## Introduction

Kawasaki disease (KD) is a vasculitis syndrome that typically affects young children under the age of five and involves multiple systems of the body. It is one of the leading causes of acquired heart diseases in children in developed countries (Newburger et al., 2016) and may lead to ischemic cardiomyopathy (Chen et al., 2016). Vascular damage during the development of KD can ultimately lead to several complications, such as coronary artery lesions (CALs), including aneurysms, and aortic root dilatation (Gordon et al., 2009). Currently, the diagnosis of KD relies on the assessment of clinical symptoms, including fever lasting five or more days, erythema of the palms and soles or edema of the hands and feet, bilateral conjunctival injection, changes in the lips and oral cavity, and cervical lymphadenopathy (McCrindle et al., 2017). Due to a lack of specific diagnostic criteria for KD, its diagnosis requires well-trained clinicians. The ability to accurately identify KD based on improved disease characterization and prognostic models may enable clinicians to make more precise treatment decisions and initiate treatment earlier, leading to a better prognosis. In KD, biomarkers could add diagnostic value to clinical features and ultrasound. Based on their represented major pathophysiologic pathways, current biomarkers are divided into the following categories: inflammation [C-reactive protein (CRP), erythrocyte sedimentation rate (ESR)], liver dysfunction [alanine aminotransferase (ALT)], and metabolic homeostasis (albumin, serum sodium) (McCrindle et al., 2017). However, it is necessary to investigate biomarkers in other pathways to fully understand the intricate pathophysiology of the condition and improve risk assessment, and the clinical need for non-invasive biomarkers to diagnose KD remains unfulfilled.

Metabolomics is an emerging, cost-effective, quantifiable tool for biomarker discovery (Johnson et al., 2016). Metabolomics can detect underlying changes in the metabolic products of physiological processes caused by cardiovascular diseases (CVDs) and provide important information on metabolic pathways and metabolites. In addition, the effects of gene mutations and environmental changes on the body are reflected by changes in metabolites. As a result, there is increasing interest in using metabolomics to define the chemical phenotypes associated with health or disease for cardiovascular risk stratification (Newgard, 2017; Ruiz-Canela et al., 2017). For example, metabolomics has revealed that the TMAO (trimethylamine N-oxide) pathway is strongly linked to myocardial infarction (Wang et al., 2011), and PAGln (phenylacetylglutamine) has been identified as a crucial prognostic factor in cardiovascular disease (Nemet et al., 2020). However, limited metabolomics studies on KD plasma have been reported, and diagnostic and prognostic applications of metabolic alterations in KD are not yet well defined; therefore, there is an urgent need to search for biomarkers and therapeutic targets for KD from a metabolomic perspective.

Tryptophan (Trp) is an essential amino acid and, as such, must be obtained from dietary sources. Dietary Trp can undergo degradation via intestinal flora (Taleb, 2019), or it can enter the bloodstream and be carried to various tissues where it is used as a substrate in various biosynthetic pathways. Trp can be used as a precursor of serotonin in the central nervous system (Ruddick et al., 2006) and as a source of the coenzyme nicotinamide adenosine dinucleotide (NAD) (Shin et al., 1998) throughout the body. The l-kynurenine (Kyn) pathway is one of the main pathways of Trp metabolism that generates Kyn and its downstream products and participates in inflammatory and immune responses (Wang et al., 2015). The Kyn pathway has been implicated in various biological processes, including immune regulation, peripheral disorders, and central nervous system disorders (Wang et al., 2015). Additionally, Trp metabolites (Kyn, anthranilic acid, and 3-hydroxy-l-kynurenine) are associated with high CVD-related mortality (Wang et al., 2010; Mangge et al., 2014). However, limited clinical data exist on the potential prognostic value of Trp pathway metabolites in patients with KD.

Transcriptomics is widely used for studying CVDs, such as atherosclerotic diseases (Herman and Autieri, 2018), hypertension (Nemecz et al., 2016), heart failure (Gao et al., 2015), and myocardial hypertrophy (Viereck et al., 2016), and has helped identify new biomarkers and therapeutic strategies. Single-cell transcriptome sequencing (scRNA-seq) technology can reveal subtle changes in each cell and describes the gene regulatory networks that alter physiological functions, behaviors, and phenotypes (Shaw et al., 2021). Aryl hydrocarbon receptor (AHR) is a ligand-activated transcription factor widely expressed in various immune cells, including T cells, dendritic cells, and intestinal intraepithelial lymphocytes. Trp metabolites have beeshown to be capable of functioning as endogenous ligands to regulate related aryl hydrocarbon receptor expression (Hezaveh et al., 2022). Combined peripheral blood single-cell transcriptome and metabolomic analyses can better verify changes in the metabolic microenvironment in plasma.

In this study, we aimed to: 1) investigate metabolic alterations in patients with KD to identify possible novel diagnostic and prognostic biomarkers for KD; 2) validate the metabolites’ change using Peripheral Blood Single-Cell Transcriptome analysis; and 3) explore changes in biomarkers in mouse KD models to support further research into therapeutic development using mouse KD models.

## Results

### Metabolite profiling of the plasma from patients with KD using untargeted metabolomics

**FIGURE 1:**
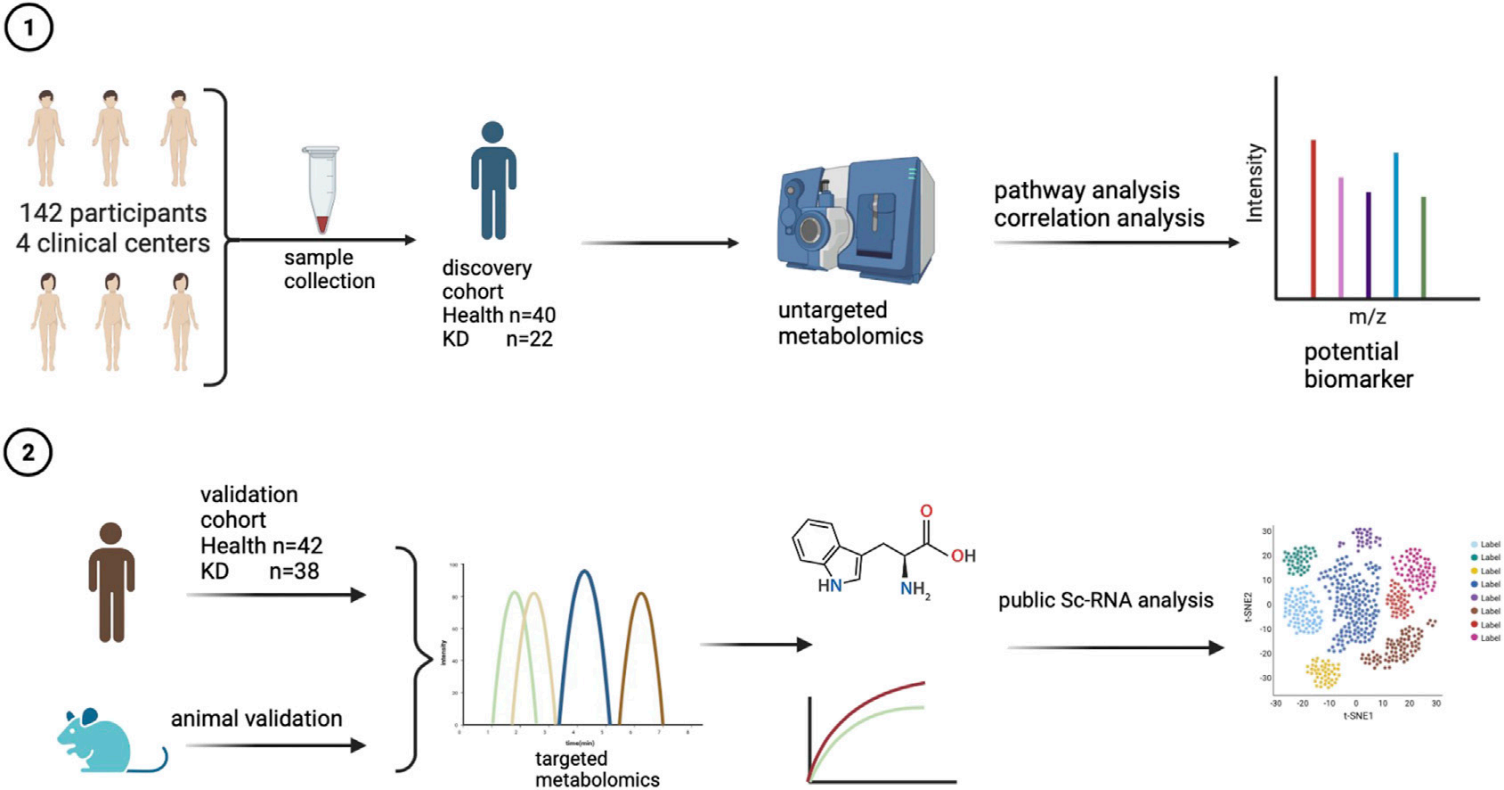
Flow chart showing the study design for metabolomics analysis and biological validation.

An overview of the workflow is presented in Figure 1. To identify potential biomarker candidates, we collected plasma samples from patients as a discovery cohort. The samples from 62 participants were subjected to untargeted metabolomics analysis using ultra-high-performance liquid chromatography-quadrupole time-of-flight mass spectrometry (UHPLC-QTOF-MS). The basic characteristics (including age and gender) of all 62 individuals were similar between the KD and healthy control groups (Table 1). However, laboratory values, including routine blood tests and biochemical indices of KD patients, exhibited a higher inflammatory state.

**TABLE 1:** Demographic and clinical characteristics of discovery cohort individuals.

	HC	KD	*p*.Overall
	*N = 40*	*N = 22*	
Age (month)	28.0 [22.0; 40.2]	29.5 [13.2; 47.8]	0.591
Gender			0.716
Female	16 (40.0%)	7 (31.8%)	
Male	24 (60.0%)	15 (68.2%)	
WBC (10^9/L)	7.08 [5.84; 8.35]	13.8 [8.91; 16.5]	<0.001
Lymphocyte ratio (%)	58.4 [45.0; 63.0]	27.6 [16.8; 31.3]	<0.001
Neutrophils (%)	31.0 [26.5; 45.3]	65.4 [55.4; 73.1]	<0.001
Platelets (10^9/L)	284 [262; 363]	392 [315; 506]	0.002
RBC (10^12/L)	4.65 (0.34)	4.20 (0.52)	0.001
Hb (g/L)	125 (7.17)	108 (9.03)	<0.001
ALT (IU/L)	15.8 [13.0; 17.0]	36.5 [16.0; 74.2]	0.001
AST (IU/L)	33.0 [29.8; 35.0]	26.5 [24.0; 31.5]	0.001
Serum albumin (g/L)	42.8 [41.9; 43.6]	36.9 [34.5; 37.7]	<0.001
Total bilirubin (μmol/L)	6.11 [4.20; 6.90]	7.65 [5.93; 8.77]	0.100

The data are reported as either the mean ± standard deviation (SD), median with interquartile range (IQR), or percentages. A two-sided *t*-test was performed on variables represented as the mean ± SD, a Wilcoxon rank sum test was performed on variables expressed as the median with IQR, and a chi-square test was performed on variables expressed as percentages. HC, health control; KD, kawasaki disease; WBC, white blood cell; RBC, red blood cell; Hb, hemoglobin; ALT, glutamic pyruvic transaminase; AST, glutamic oxaloacetic transaminase.

Untargeted metabolomics was performed using ultra-performance liquid chromatography coupled with tandem mass spectrometry (UPLC-MS/MS) detectors. After instrumental analysis, peak detection, and alignment, 2,345 mass features in negative electrospray ionization (ESI–) and 2,645 in positive ESI+ were detected in the discovery cohort, 530 serum small-molecule metabolites were identified, and the annotated data matrices were used for further statistical analysis.

**FIGURE 2:**
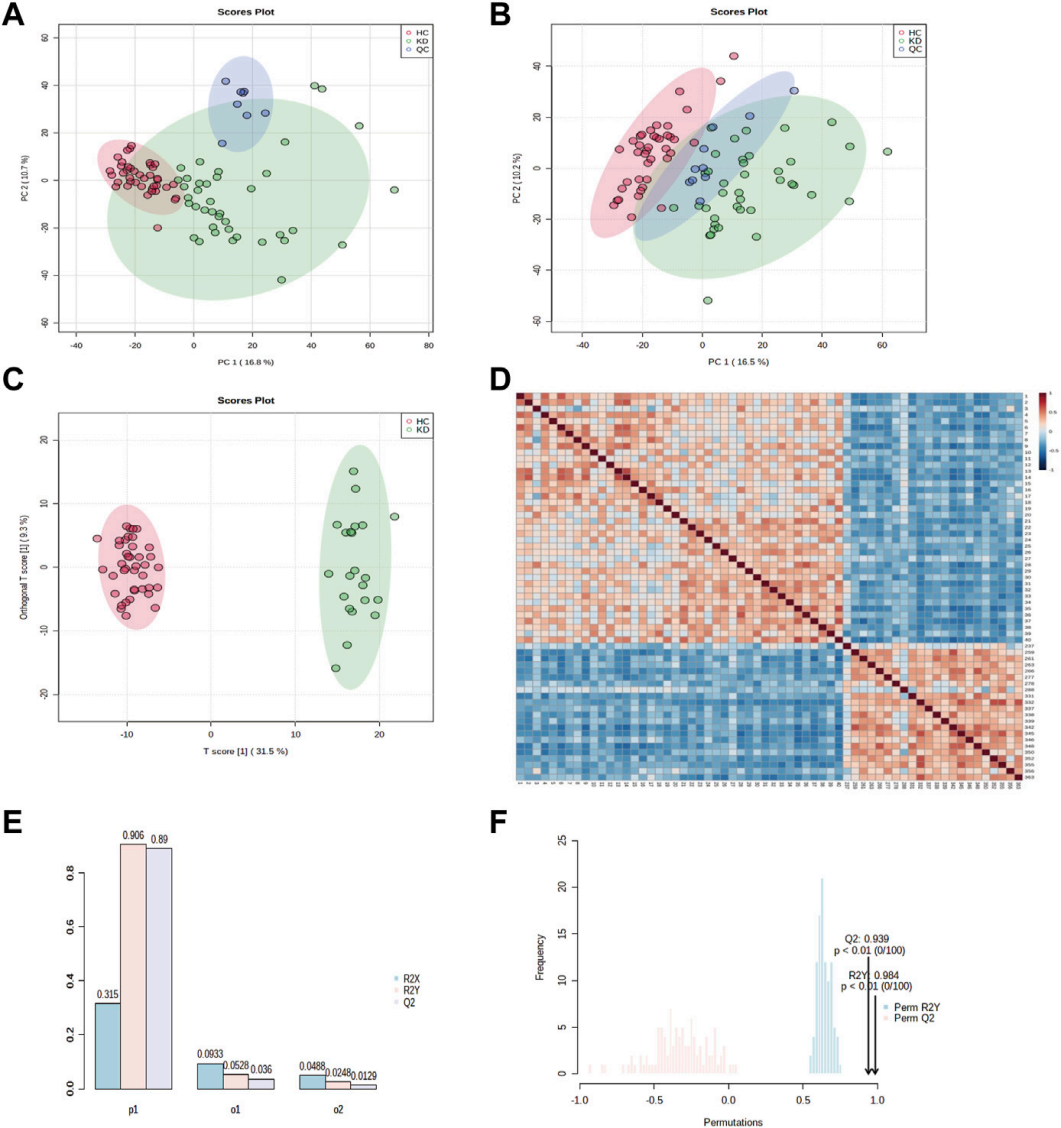
Principal component analysis (PCA) classification models for KD and HC in the discovery cohort. PCA score plots for patients with KD and healthy controls in **(A)** electrospray (ESI)+ mode and **(B)** ESI–mode. Healthy controls (HCs) are marked in red, patients with KD are marked in green, and quality control (QC) samples are shown in blue. The *x* and *y* axes represent the contributions of individuals to the first two principal components, PC1, and PC2, respectively. The OPLS-DA score plot shows the separation between patients with KD and healthy controls for all metabolites **(C)**. The heat map shows the change in the abundance of metabolites in the plasma of healthy controls (*n* = 40) and patients with KD (*n* = 22) **(D)**. Overview of the OPLS-DA model showing the *R* ^2^X, *R* ^2^Y, and *Q* ^2^ coefficients for the groups **(E)**. Permutation analysis of *R* ^2^Y and *Q* ^2^ coefficients repeated 200 times **(F)** (*Q* ^2^ = 0.939 and *R* ^2^Y = 0.984).

First, ESI- and ESI + quality control (QC) samples were used to build principal component analysis (PCA) models to assess the quality of the metabolomics data. The QC samples clustered tightly together in both negative and positive modes, illustrating the high stability and reliability of the data (Figures 2A,B). In addition, the PCA distinguished clusters of samples from the two groups. To maximize the identification of differential metabolites in patients with KD, we constructed orthogonal projections to latent structures (OPLS-DA model) to observe the main discriminations of metabolomics between the two groups and identify significantly altered metabolites (Figure 2C). Next, we evaluated the performance of the model in correctly categorizing new samples using 7-fold cross-validation and 100 random permutation tests. The *R* ^2^ and *Q* ^2^ goodness-of-fit intercepts indicated that the OPLS-DA model was accurate and did not exhibit overfitting (Figures 2D,E).

The sample correlation heatmap showed that the samples between groups were scattered, and the samples within groups were significantly correlated (Figure 2F). The PCA, OPLS-DA, and the heatmap showed that KD patients were separated from healthy controls and had different metabolic signatures compared with those of the healthy controls.

**FIGURE 3:**
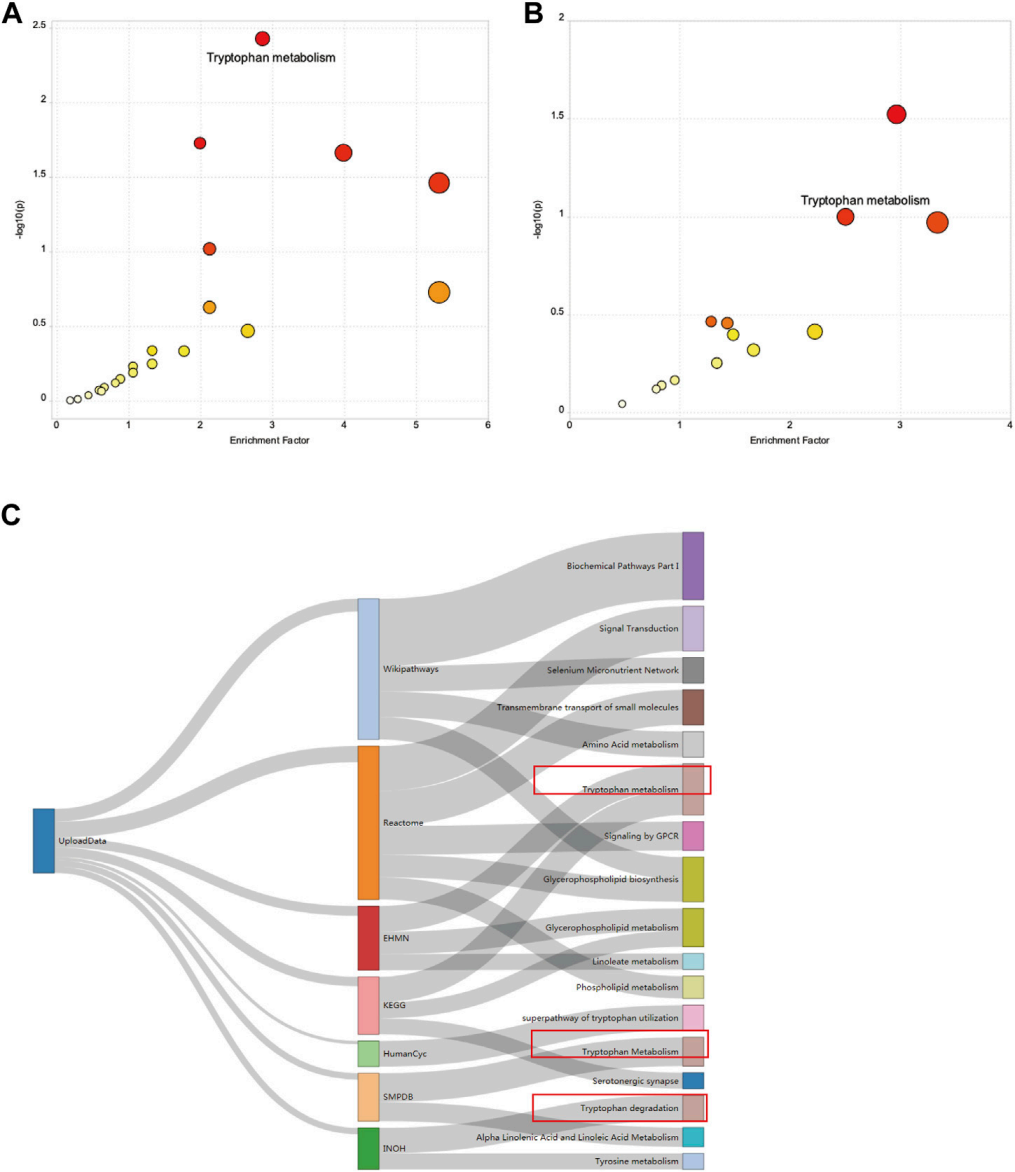
Results of the pathway enrichment analysis. A bubble chart showing the significantly enriched pathways between HCs and patients with KD from **(A)** electrospray (ESI)+ mode and **(B)** ESI- mode using Mummichog analysis before metabolite annotation. The *x* and *y* axes represent the enrichment factor of the pathway and the negative log 10 of the *p*-value, respectively. A Sankey diagram showing the altered metabolic pathway identified based on seven different databases **(C)**.

**FIGURE 4:**
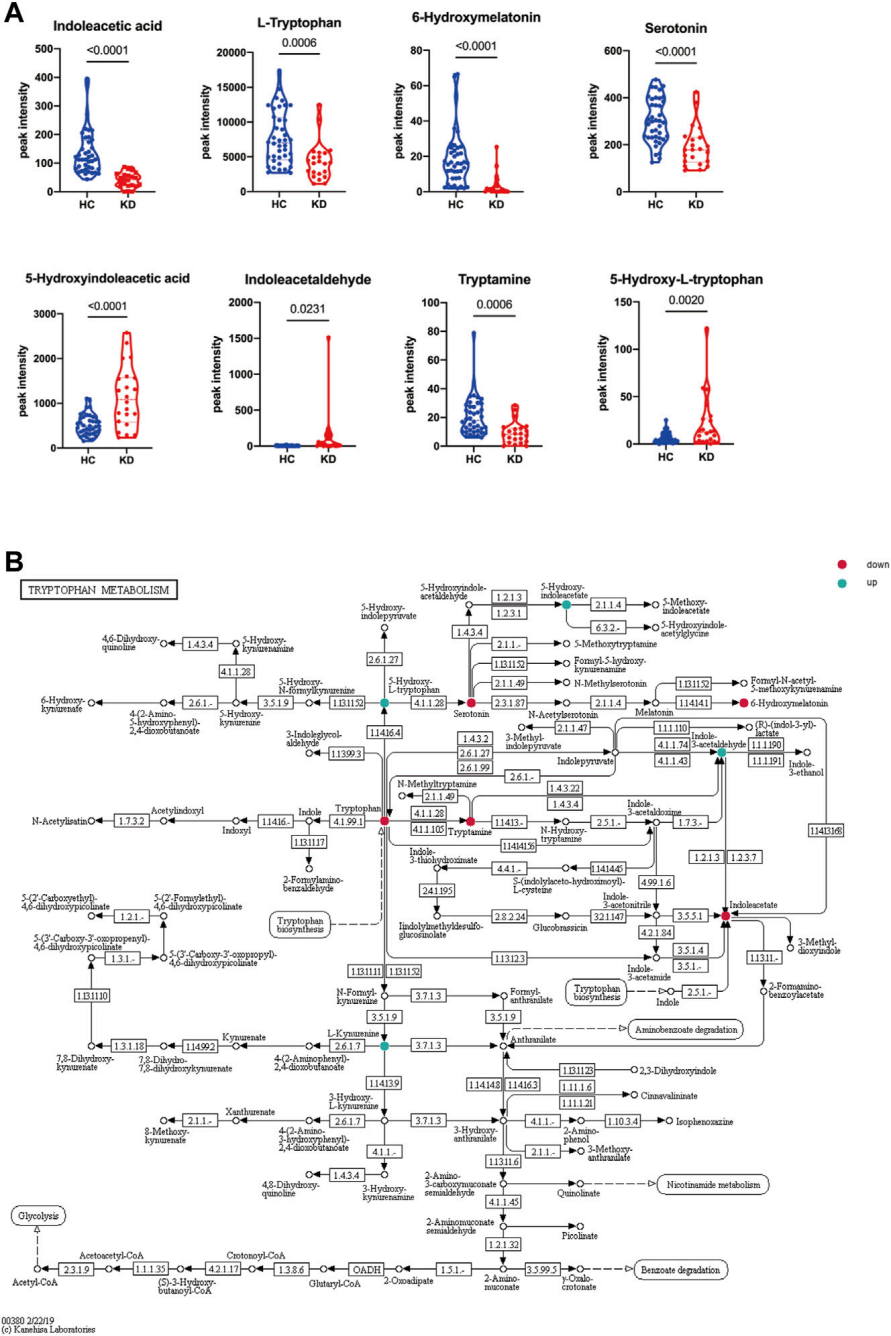
Altered tryptophan (Trp) metabolism in patients with KD. Relative abundance of Trp-related metabolites in the discovery cohort. Blue indicates HCs, and red indicates patients with KD **(A)**. The Trp metabolic pathway was visualized by a KEGG diagram, and differentially expressed metabolites are circled **(B)**. Red indicates downregulated metabolites, and blue indicates upregulated metabolites. Data are represented as the mean ± standard error (SE). Statistical analysis was performed using two-tailed Student’s *t*-tests.

To investigate alterations in the metabolic pathways of all m/z features in both positive and negative ion modes, a Mummichog pathway analysis was performed. The detailed pathway enrichment results can be seen in Supplementary Tables S1, 2. Tryptophan metabolism pathway (adjp = 0.00083892) was the most drastically enriched pathway for positive-mode data using the mummichog analysis approach. Tyrosine metabolism (adjp = 0.00089019), phenylalanine metabolism (adjp = 0.0012113), glycerophospholipid metabolism (adjp = 0.0057432), and fatty acid biosynthesis (0.019335) were also significantly enriched in positive ion mode enrichment. Porphyrin and chlorophyll metabolism (adjp = 0.00093957) and tryptophan metabolism (adjp = 0.0012581) were the top two differently enriched pathways for negative-mode data using the mummichog analysis approach. Biosynthesis of unsaturated fatty acids (adjp = 0.0036836), linoleic acid metabolism (adjp = 0.023143), and tyrosine metabolism (adjp = 0.027834) also showed significantly differential enrichment in negative ion pattern analysis. The analysis revealed that Trp metabolism was notably enriched in both ESI+ (*p* = 0.0008) and ESI– (*p* = 0.0012) modes (Figures 3A,B). Furthermore, annotations based on accurate mass and tandem MS fragmentation data were subjected to traditional pathway analyses. To reduce the bias induced by pure bioinformatic analysis, we used multiple databases to increase the reliability, including SMPDB, INOH, KEGG, REACTOME, and EHMN. These aforementioned pathways, including amino acid metabolism, tyrosine metabolism, and linoleate metabolism, were also validated with the analysis using multiple databases (Figure 3C). In the discovery cohort, the relative abundance of Trp-related metabolites are presented in Figure 4A, while Figure 4B shows the differentially expressed metabolites visualized through a KEGG diagram.. Pathway analysis revealed that these integrated results were generally consistent with the results of Mummichog analysis, indicating that patients with KD have significantly altered Trp metabolism.

### Trp-targeted metabolomic profiles in patients with KD and mice with LCWE-induced KD coronary arteritis

**FIGURE 5:**
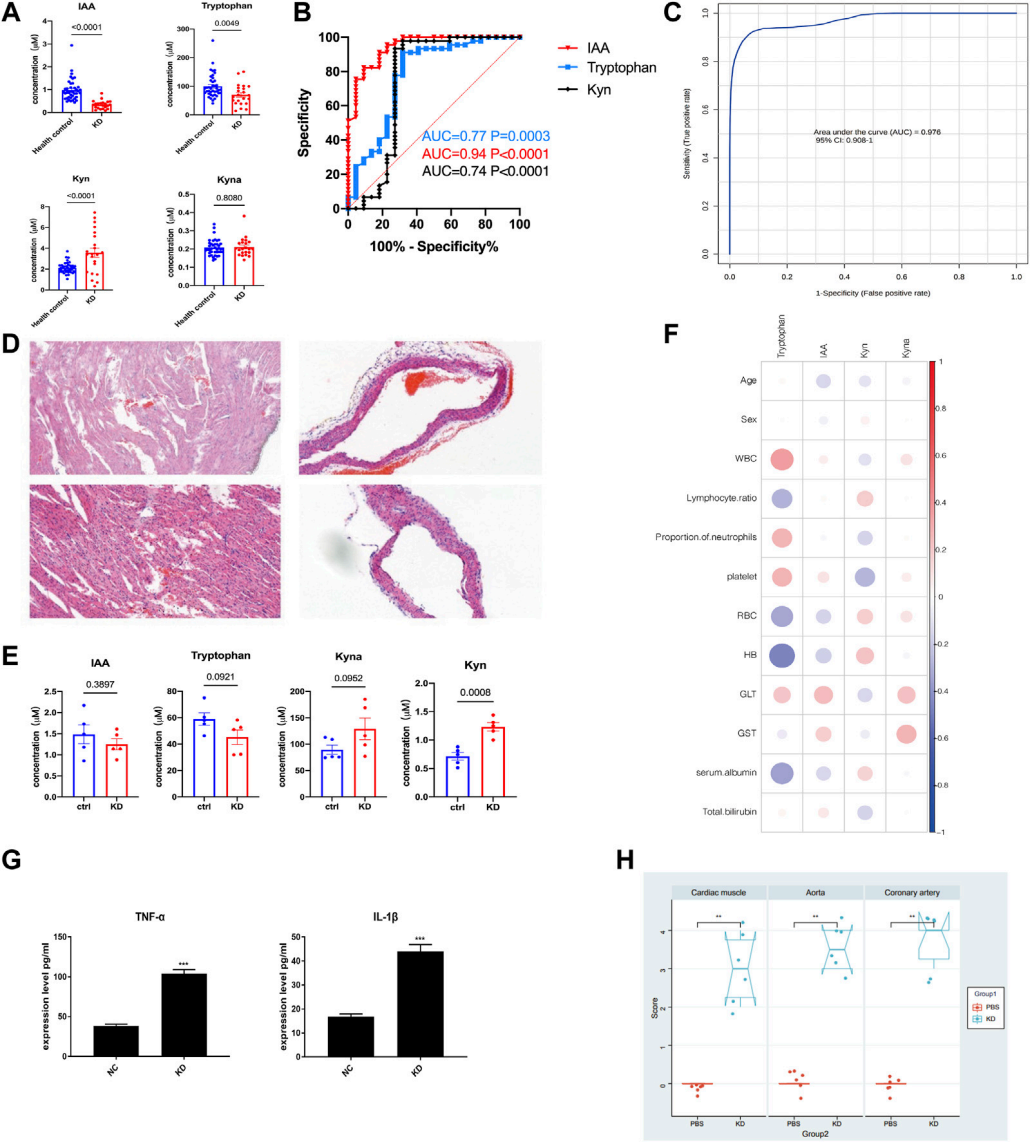
The LCWE mouse model and internal validation of metabolic alterations in patients with KD. Bar plots showing the serum levels of Trp-related metabolites in the discovery cohort (HC, *n* = 40; KD, *n* = 22) **(A)**. Receiver operating characteristic (ROC) curve showing the discrimination accuracy of the three metabolites in the discovery cohort **(B)**. ROC analysis incorporating indole acetic acid (IAA), Trp, l-kynurenine (Kyn), and kynurenic acid (Kyna) **(C)**. Hematoxylin and eosin (HE) staining of myocardial tissue of the KD mouse model compared with that of the controls **(D)**. Serum levels of IAA, Trp, Kyn, and Kyna in the KD mouse model **(E)**. Correlation coefficients between serum metabolite levels and clinical characteristics in the validation cohort **(F)**. Data were analyzed using Spearman’s rank correlation test. Levels of the pro-inflammatory cytokines, tumor necrosis factor (TNF)-α and interleukin (IL)-1β, in the serum of the LCWE mouse model **(G)**. Myocardial inflammation and heart vessel inflammation scores for each group **(H)**. Data are represented as the mean ± SE. Statistical analysis was performed using two-tailed Student’s *t*-tests **(A, E, (G)** and two-tailed Mann–Whitney U-tests **(H)**. CI, confidence interval. *, *p* < 0.05; **, *p* < 0.01; ***, *p* < 0.001.

Untargeted metabolomic profiling demonstrated notable changes in the Trp metabolic pathway within the plasma of KD patients, suggesting that Trp metabolism may play an important role in the occurrence and development of KD. To confirm these results, serum levels of Trp, Kyn, IAA, and Kyna were quantified using a fully validated LC-MS/MS-based targeted metabolomics method, and the results were consistent with those of untargeted metabolomics. In patients with KD, compared with healthy subjects, there were significantly lower levels of Trp (65.6 µM vs 88.0 µM; *p* = 0.0003) and IAA (0.61 µM vs 1.18 µM; *p* < 0.0001), whereas the Kyn level was significantly higher (3.97 µM vs 2.45 µM; *p* < 0.0001; Figure 5A). Additionally, IAA, Kyn, and Trp in patients with KD compared with those in healthy subjects had a higher area under the curve (AUC) values (0.94, *p* < 0.0001; 0.77, *p* = 0.003; and 0.74, *p* = 0.001, respectively; Figure 5B). The combination of IAA, Trp, Kyn, and Kyna in the metabolite panel had a high AUC value (0.979, 95% CI 0.92–1; Figure 5C). Additionally, a correlation analysis of these differential metabolites and clinical characteristics was performed to better understand the relationship between the Trp metabolism panel and KD. Trp levels were only negatively associated with hemoglobin levels, indicating that the metabolism panel differs from that of existing biomarkers, such as clinical biochemical indices (Figure 5F and Supplementary Table S1).

After considering the relevance of the mouse model of KD to human disease, we used a mouse model to further examine the Trp pathways to provide evidence for identifying potential biomarkers and targeted discoveries from studies on the discovery cohort. We built a *Lactobacillus casei* cell wall extract (LCWE) induced KD model; the model was shown to be successfully constructed by Hematoxylin and eosin (HE) staining, inflammation scores, and serum cytokine detection.

HE staining of myocardial tissue showed significant inflammatory cell accumulation and bleeding manifestations in the mice in the KD group (Figure 5D). Compared with the control group, the KD group exhibited significantly dilated CALs with a high concentration of infiltrating inflammatory cells, and the inner lining of the blood vessels appeared less smooth. Serum TNF-α and IL-1β levels were significantly higher in the KD mice than those in the control mice (Figure 5G; *p* < 0.0001). The mice in the KD group also had significantly higher inflammation scores than those in control mice (Figure 5H; *p* < 0.05), indicating the successful creation of the KD coronary arteritis mouse model. Through targeted metabolomics focusing on Trp metabolism, we studied the changes in potential biomarkers in the plasma of mice with KD. Consistent with the observations in patients with KD, Kyna, and Kyn levels were increased, whereas Trp and IAA levels were reduced in these mice (Figure 5E).

### External validation of altered trp metabolites in the validation cohort

**FIGURE 6:**
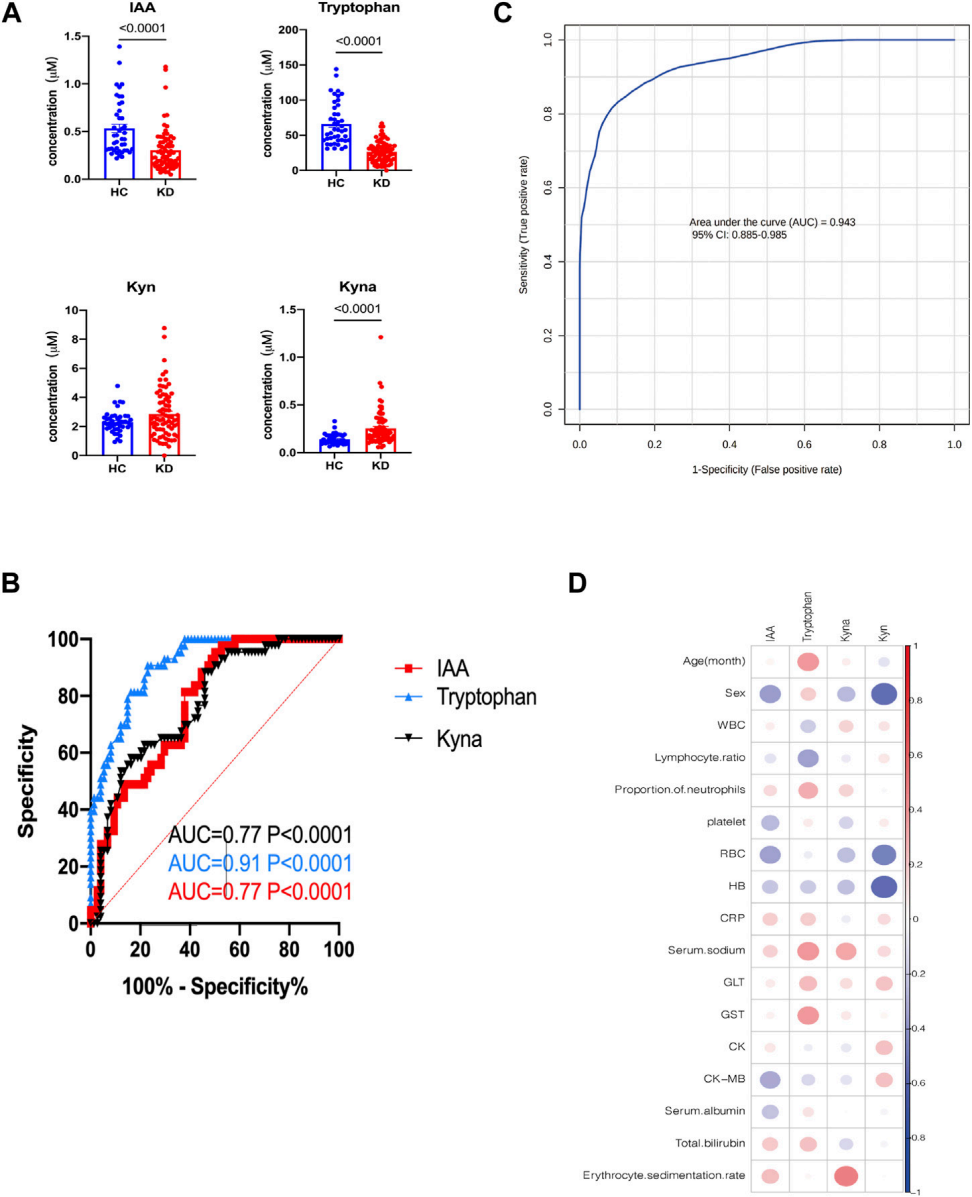
External validation of potential biomarkers for patients with KD. Bar plots showing the serum levels of Trp-related metabolites in the validation cohort (HC, *n* = 42; KD, *n* = 38) **(A)**. Receiver operating characteristic (ROC) curve showing the discrimination accuracy of Trp-related metabolites in the validation cohort **(B)**. Data represent the mean ± SE. The *p*-value was calculated using two-tailed U-tests. ROC analysis incorporates IAA, Trp, Kyn, and Kyna in the validation cohort **(C)**. Correlation coefficients between metabolite serum levels and clinical characteristics in the validation cohort **(D)**. Association strength was assessed using Spearman’s rank correlation test. IAA, indole acetic acid; Kyn, l-kynurenine; Kyna, kynurenic acid.

To further validate the metabolic changes regarding Trp metabolism in patients with KD and identify potential biomarkers, we enrolled another set of 38 patients with KD and 42 healthy controls (validation cohort) for targeted metabolomic analysis and verification of potential biomarkers. The clinical characteristics of the validation cohort participants are presented in Table 2. There were no notable differences between the two groups regarding sex and age. In this investigation, the profiles of Trp and its metabolites again exhibited apparent separation between the KD patients and healthy controls. The direction of changes in Trp-related metabolites was consistent with previous observations in the discovery cohort. Patients with KD had lower Trp (26.2 µM vs 57.5 µM, *p* < 0.0001) and IAA levels (0.24 µM vs 0.42 µM, *p* < 0.0001) but higher serum Kyna levels (0.19 µM vs 0.12 µM, *p* < 0.0001) compared with those of control subjects (Figure 6A). No marked difference existed in the Kyn levels between the two groups in the validation cohort, whereas IAA and Trp exhibited concentration level shifts in directions concordant with the results of the discovery dataset. The relative ratio of downstream Kyn metabolism to Trp metabolism was increased in both cohorts (discovery cohort, *p* = 0.0002; validation cohort, *p* < 0.0001).

**TABLE 2:** Demographic and clinical characteristics of validation cohort individuals.

	HC	KD	*p*.Overall
	*n = 42*	*n = 38*	
Age (month)	29.0 (12.1)	24.6 (15.1)	0.161
Sex			1.000
Female	20 (47.6%)	19 (50.0%)	
Male	22 (52.4%)	19 (50.0%)	
WBC (10^9/L)	8.74 (2.07)	13.5 (3.24)	<0.001
Lymphocyte ratio (%)	33.0 (10.5)	25.9 (11.0)	0.004
Neutrophils (%)	51.0 (8.07)	67.4 (10.4)	<0.001
Platelets (10^9/L)	323 [276; 362]	463 [395; 524]	<0.001
RBC (10^12/L)	3.84 [3.50; 4.48]	4.07 [3.87; 4.26]	0.541
Hb (g/L)	104 [96.0; 111]	110 [105; 114]	0.006
Serum sodium (mmol/L)	140 [138; 142]	134 [132; 135]	<0.001
ALT (IU/L)	26.0 [19.5; 34.5]	65.5 [39.5; 86.5]	<0.001
AST (IU/L)	25.5 [15.2; 34.0]	32.0 [21.2; 54.0]	0.006
Serum albumin (g/L)	24.6 [21.6; 26.6]	35.8 [33.7; 36.8]	<0.001
Total bilirubin (μmol/L)	6.75 [5.23; 9.88]	5.40 [4.50; 7.97]	0.025

Data are presented as the mean ± standard deviation (SD) and median with interquartile range (IQR). The *p*-values are based on the two-sided *t*-test for variables expressed as the mean ± SD, Wilcoxon rank-sum test for variables expressed as median (IQR), and chi-square test for variables expressed as percentages. HC, health control; KD, kawasaki disease; WBC, white blood cell; RBC, red blood cell; Hb, hemoglobin; ALT, glutamic pyruvic transaminase; AST, glutamic oxaloacetic transaminase.

Additionally, receiver operating characteristic (ROC) curve analysis showed that IAA, Trp, and Kyna had high AUC values of 0.77, 0.91, and 0.94, respectively (Figure 6B). The combination metabolite panel, including IAA, Trp, Kyn, and Kyna, had a higher AUC value of 0.943 (Figure 6C). Similarly, the levels of these different metabolites were not associated with routine blood tests or biochemical indices (Figure 6D, Supplementary Table S2).

### Expression of a Trp metabolism-related gene in peripheral blood mononuclear cells of patients with KD

**FIGURE 7:**
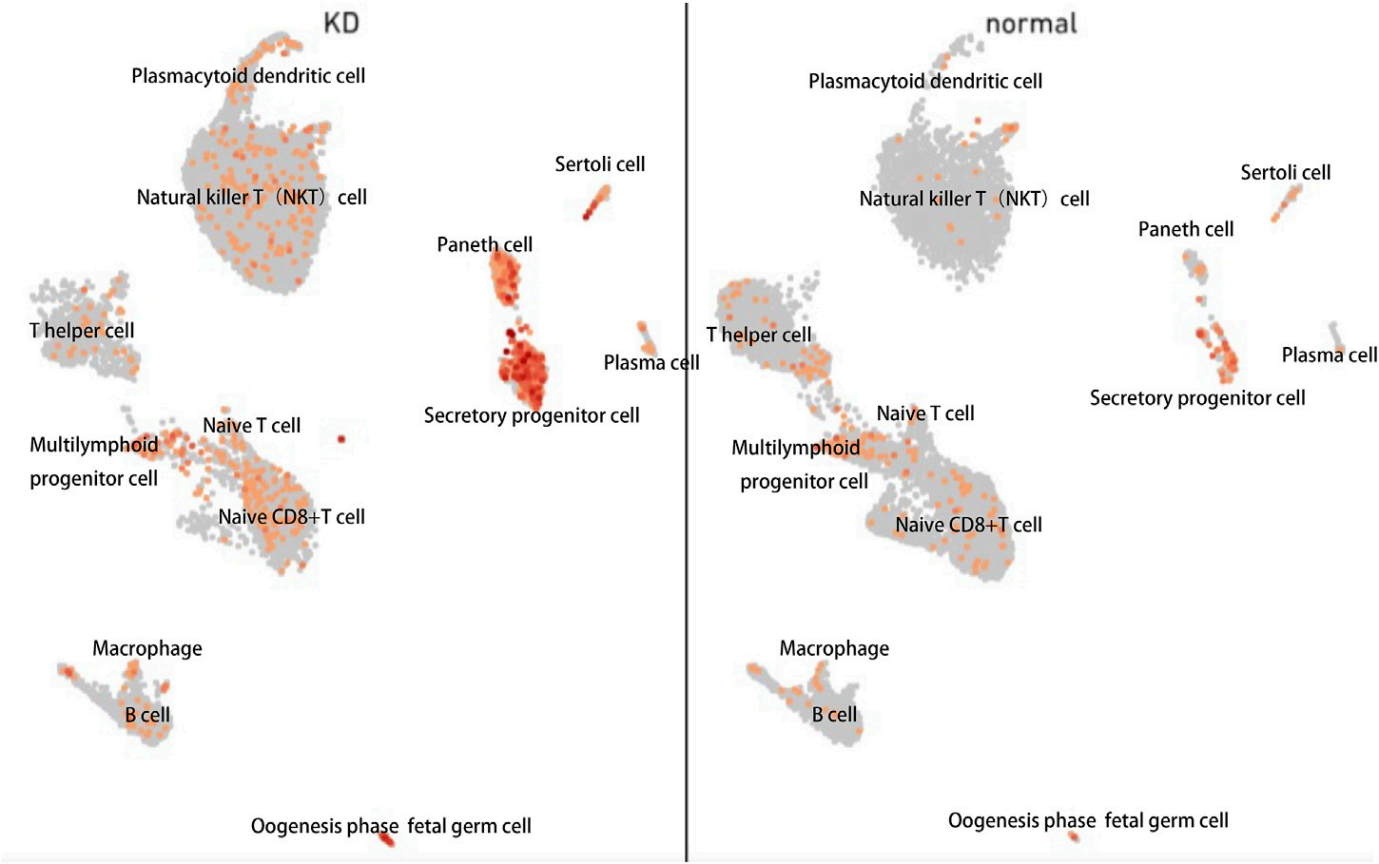
Single-cell (sc)-RNA-sequencing analysis of peripheral blood mononuclear cells harvested from patients with KD revealed increased aryl hydrocarbon receptor levels. Each point represents a cell. Points with close spatial distance indicate that the gene expression patterns of these cells are relatively close, such that the expression patterns of cells in the same cluster were most similar.

The Aryl hydrocarbon receptor (AHR), a transcription factor that depends on ligands, is expressed extensively in epithelial, endothelial, and immune cells. In previous studies, cell groups were clustered into 12 cell clusters in two samples, one from a healthy child and the other from a patient with KD. Compared with the healthy child, the KD patient had low levels of naive CD8^+^ T cells, T helper cells, and B cells; conversely, the number of immune-related T cells and natural killer T (NKT) cells was higher in the KD patient. We reanalyzed public single-cell RNA sequencing (scRNA-Seq) data from our previous study on peripheral blood mononuclear cells (PBMCs) from patients with KD (Fan et al., 2021). By comparing the gene expression of cells in each cluster and all remaining cells, specific marker genes of the cluster were identified, and the expression of aryl hydrocarbon receptor was found to be significantly changed in most PBMC types, including NKT, secretory progenitor, and plasmacytoid dendritic cells, between patients with KD and healthy individuals, as indicated by the lattice heatmap (Figure 7). The measured changes in aryl hydrocarbon receptor expression levels were consistent with the increased levels of endogenous aryl hydrocarbon receptor ligands, such as Kyn, in the serum.

## Discussion

KD is a self-limiting systemic vasculitis that predominantly affects medium-sized arteries (Nakamura, 2018). The development of KD mainly manifests as chronic inflammation caused by immune cell infiltration and progressive remodeling of vascular tissue (Johnson et al., 2016), and its diagnosis is currently based on clinical symptoms. There is currently no recommended blood-based biomarker for diagnosing KD in medical guidelines.

A few studies have proposed the use of proteins or additional inflammatory parameters as potential biomarkers for KD. For example, Zandstra *et al.* reported the use of C-reactive protein (CRP), myeloid-related protein 8/14 (MRP8/14 or S100A8/9), and human neutrophil-derived elastase (HNE) for discriminating KD from infectious diseases (Zandstra et al., 2020). In another study, urine proteomic analysis revealed 43 differentially expressed proteins between patients with KD and normal controls, including serine hydroxy-methyltransferase 1, which was regarded as a hub protein (Hu et al., 2019). Based on routine laboratory tests, serum ferritin may be a useful biomarker to distinguish KD from other acute febrile illnesses (Kim et al., 2021). Moreover, it has been suggested that IFN-γ-inducible protein 10 (IP-10) can facilitate the early diagnosis of KD (Ko et al., 2015). Metabolic profiling is emerging as an efficient approach for detecting different diseases that are not easily diagnosed. However, there are limited studies exploring KD from a metabolome perspective. In the current study, we recruited a discovery and a validation cohort, established animal models of KD, and integratedly analyzed the metabolome profile shift of KD using untargeted metabolomics and targeted metabolomics.

Our metabolomic analysis revealed an underlying metabolic signature in the plasma of patients with KD. We first performed pathway enrichment in the discovery cohort from MS peaks using the well-established Mummichog. We then annotated the metabolites and comprehensively applied a variety of databases to conduct pathway analysis on the identified metabolites. The pattern of metabolic abnormalities that we found in the phospholipid oxidation pathway was consistent with that reported in a recent study by Nakashima and others (Nakashima et al., 2021). Lipid abnormalities appear in many immune disorders and different phases of the inflammatory process, such as rheumatoid arthritis (Steiner and Urowitz, 2009) and type-1 diabetes (O’Brien et al., 1998). We also observed amino acid metabolism pathways, including those of tyrosine that were enriched with metabolites that differed between KD and healthy subjects. Alterations in amino acid metabolism are widespread in metabolic disorders and participate in the immune response caused by pathogen infection (Tomé, 2021). Among these altered metabolic pathways, the tryptophan pathway was the most significantly enriched pathway. The serum concentrations of Trp pathway metabolites were measured using targeted metabolomics because it is a more quantitatively sound approach with greater clinical utility than untargeted metabolomic assessments. The results of further targeted detection of metabolites of the tryptophan pathway replicated the non-targeted analysis results. The prognostic metabolites that were found to discriminate patients with KD from healthy individuals were from the Trp pathway, including Trp itself, IAA, Kyn, and Kyna. Tryptophan is one of the several amino acids that are essential in mammals and acts as a precursor of many signaling molecules that regulate adaptive immune responses (Liu et al., 2017). It exhibits the highest antiradical activity among all amino acids in cellular proteins. Weiss et al. suggested that Trp is a potent scavenger of the radicals that are induced by chloramine-T or hydrogen peroxide (Weiss et al., 2002). The decrease in Trp levels in patients with KD may contribute to the progression of KD symptoms, as supplementation of Trp has been shown to be beneficial (Gibson, 2018). The primary catabolic pathway of Trp in mammals is the Kyn pathway, which involves the constitutive catalysis of Trp to Kyn by three key rate-limiting enzymes—indoleamine 2,3-dioxygenase 1 and 2 (IDO1 and IDO2) and Trp 2,3-dioxygenase (Ketelhuth, 2019). Kynurenine is important to the pathogenesis of aortic diseases by contributing to inflammation in various vascular beds (Ramprasath et al., 2021). Studies have shown that Kyn exhibits pro-oxidant effects when exposed to aerobic radiation, resulting in the production of superoxide radicals, which can lead to the reduction of cytochrome C (Goda et al., 1987). Increased levels of Kyn can cause NKT cell death mediated by reactive oxygen species (ROS) (Song et al., 2011). This indicates that treatments targeting Kyn may be useful for patients with oxidative stress-related diseases. Kynurenic acid is an intermediary in the Trp metabolic pathway and functions as a ligand for the orphan G protein-coupled receptor 35 (Wang et al., 2006). It can be generated by kynurenine aminotransferases under physiological conditions in endothelial cells (Stazka et al., 2002) and human PBMCs (Jones et al., 2015). Endothelial dysfunction is a critical process implicated in the development of KD. Indole acetic acid, which is derived from Trp, has been shown to decrease inflammation and ROS (Ji et al., 2020) by reducing the expression of pro-inflammatory cytokines (Shen et al., 2010). In the present study, the decreased concentration of IAA may be due to KD development. Overall, the identified metabolites and pathways fit the pathophysiological profile of KD.

Despite the high variability in the blood metabolome between and within individuals, the metabolic signature we constructed herein performed acceptably as a diagnostic tool for KD in two independent cohorts, with AUCs of 0.976 and 0.943. In a previous study, good intra-group repeatability was observed for the results of Kyn, Kyna, xanthurenic acid, 3-hydroxy-l-kynurenine, anthranilic acid, 3-hydroxyanthranilic acid, and the Kyn/Trp ratio in samples obtained from both the chronic heart failure and control groups, supporting the use of Trp pathway metabolites as biomarkers (Kato et al., 2010). Fortunately, KD and heart failure are uncommon, and these diseases rarely overlap.

Next, we investigated the metabolic changes of Trp metabolism in the LCWE-induced KD coronary arteritis mouse model. Consistent with our hypotheses, Trp metabolism and the correlated metabolites trended in a similar pattern as those in patients with KD. Integrating the cross-species data from humans and mice may provide more opportunities for identifying potential biomarkers and making targeted discoveries.

There are numerous interactions between circulating metabolites in plasma and blood cells. For instance, Kyn mediates the activation of aryl hydrocarbon receptor, a ligand-activated cytoplasmic receptor and transcription factor capable of reinforcing the inflammatory state by boosting the production of interleukin-6 (Guarnieri, 2022). Hence, we performed data mining on a typical pediatric PBMC single-cell transcriptome and found that aryl hydrocarbon receptor expression was significantly altered between children with KD and healthy subjects. The stimulated Kyn metabolic pathway and aryl hydrocarbon receptor may indicate increased ligand-receptor interactions between Kyn and aryl hydrocarbon receptor. The downstream aryl hydrocarbon receptor expression changes are consistent with changes in the Trp pathway, which verified the reliability of our results—the tryptophan metabolic pathway is indeed altered in KD patients. In addition, the change in the tryptophan pathway can be used not only as a phenotype but may also play a crucial role in the regulation of the pathological development of KD by affecting transcription factors such as aryl hydrocarbon receptor. We therefore speculate on the therapeutic utility of Trp pathway metabolites in KD, but further biochemical experiments are needed to verify this assumption Notably, increased IDO activity in the serum has been observed in patients with advanced atherosclerosis, indicating that activated kynurenine pathway may play a pivotal role in the development of vascular diseases (Ji et al., 2020). On the other hand, in the kynurenine pathway, IDO1 is the primary rate-limiting enzyme. Furthermore, several recent studies have demonstrated that the expression of IDO1 is elevated in response to inflammatory stimuli, such as type I and II interferons (Puccetti and Grohmann, 2007), prostaglandins (Jones et al., 2015), or microbial stimuli, such as lipopolysaccharides (Michaux et al., 2022). Meanwhile, IDO1 is regarded as a target gene to regulate overactive immune responses in human autoimmune diseases (Pan et al., 2008; Platten et al., 2012; Kasper et al., 2016).

Our results suggest that the Trp metabolic pathway is significantly altered in KD, particularly Trp itself, IAA, Kyn, and Kyna. Single-cell transcriptome analysis results corroborated metabolomic results to some extent. These metabolic indicators may serve as novel biomarkers and help in developing new strategies for the diagnosis and treatment of KD. Interventions in specific microorganisms targeting microbiota-ido1-aryl hydrocarbon receptor axis modulation in the host may offer innovative therapeutic strategies for treating KD.

Our study suggests that four metabolites–Trp, Kyn, Kyna, and IAA–are significantly altered in patients with KD and an LCWE-induced coronary arteritis mouse model, and that they may be potential biomarkers for diagnosing KD. However, a potential weakness of our study is that expression of the Kyn pathway increased during extensive inflammatory conditions, and it cannot be ruled out that some of the differences in metabolic profiles may be due to systemic inflammation. Thus, we should study more inflammatory disorder cohorts, such as those with measles, COVID-19, and scarlet fever, to better assess the specificity of this biomarker panel to differentiate KD. Moreover, we did not determine whether these altered levels of metabolites contribute to the inflammatory process in KD. Based on the combined metabolic analysis, we speculate that these metabolites may play key roles in the genesis and pathological development of the disease. Further studies are needed to determine the underlying mechanisms and elucidate whether these metabolites can predict the risk of KD. Furthermore, larger and well-characterized patient cohorts are needed to validate our study.

## Materials and methods

### Human participants

A total of 142 participants from four clinical centers, including 82 healthy children and 60 KD patients, were recruited from Shenzhen Children’s Hospital, Longgang District Maternal & Child Healthcare Hospital of Shenzhen City, The First Affiliated Hospital of Jinzhou Medical University, and Fushun Mining Bureau General Hospital, China, from January to October 2021. Sixty-two and eighty participants were included in the discovery and validation cohorts, respectively (Figure A). The clinical characteristics of the patients are summarized in Table 1 and Table 2. The diagnostic criteria were based on the American Heart Association guidelines for KD from 2017 (Johnson et al., 2016). The exclusion criteria included children who were not at the initial stage of the disease or had a course of disease >10 days, children with other congenital heart malformations, and those who had received treatment before admission. This study followed the guidelines of the Declaration of Helsinki and received approval from the Ethics Committees of Shenzhen Children’s Hospital (protocol code 202003802) and Shenzhen Longgang Maternal & Child Healthcare Hospital (LGFYYXLLL-2022-004).

### Mice and treatments

C57/WT mice (four-to five-week-old) were purchased from the Hunan Saike Jingda Experimental Animal Company (Changsha, China). The mice were intraperitoneally injected with a single dose of 400 µg of LCWE (day 0) to induce KD vasculitis; LCWE was prepared as previously described (Porritt et al., 2020). The mice were euthanized 14 days post-LCWE injection, and their hearts were extracted. Mouse tissues were collected for further histopathological examination, and serial sections (4 μm) were made and stained with HE. Stained sections were photographed using a fluorescence microscope (Olympus, Tokyo, Japan). Inflammation scores for coronary arteritis, aortitis, and myocarditis were assessed to evaluate the severity of inflammation. The serum levels of IL-1β and TNF-α were determined by enzyme-linked immunosorbent assay.

### Chemicals and materials for metabolomic analysis

High-performance liquid chromatography (HPLC) grade acetonitrile, methanol, formic acid was purchased from Thermo Fisher Scientific, Waltham, MA, United States, and liquid chromatography–mass spectrometry (LC–MS) grade water was purchased from A.S. Watsons Group Co., Hong-Kong, China. Unlabeled standards for targeted metabolomics were purchased from Cayman Chemical (Ann Arbor, MI, United States). The labeled standards, d4-IAA and d5-tryptophan, were purchased from Toronto Research Chemicals (Toronto, ON, Canada). All reagents and chemicals were of the highest purity (>99%) and stored at appropriate temperatures and conditions.

### Sample collection

Blood samples were collected in heparinized tubes according to standard procedure, followed by centrifugation at 3,000 rpm at 4°C for 10 min to obtain plasma. The plasma was then aliquoted and stored at -80°C within 2 h of collection. The samples were transported to the lipid center at Capital Medical University and kept there until the end of recruitment.

### Sample preparation

The plasma samples were first mixed with three volumes of acetonitrile and incubated to precipitate proteins. The supernatant was collected after centrifugation and then evaporated under a vacuum to remove the solvent. Thereafter, the dried extracts were resuspended in a mixture of acetonitrile and water, vortexed, and centrifuged again to remove any remaining particles. Subsequently, the recovered supernatant was subjected to LC–MS analysis, and samples were injected in a randomized order during the runs to prevent bias. A quality control (QC) sample, which was a mixture of all plasma samples, was injected between every 10 sample injections to monitor consistency in the retention time and signal intensity.

### Mass spectrometry

Metabolic extracts were analyzed using reversed-phase liquid chromatography–mass spectrometry (RPLC-MS) in both positive and negative electrospray ionization modes. An AB SCIEX Triple TOF 5600 mass spectrometer (SCIEX, Framingham, MA, United States) was used to acquire data from 50 to 1,000 m/z in a 0.25 s TOF-MS scan mode. MS/MS spectra of the quality control (QC) sample was obtained using an information-dependent acquisition (IDA) method; the parameters were as follows: ion spray voltage, 5,500 V (+) and 4,500 V (−); interface heater temperature, 550°C (+) and 600°C (−); curtain gas of 35 PSI; declustering potential, 100 V (+) and −100 V (−); collision energy, 10 eV (+) and −10 eV (−). The range of m/z was set to 25–1,000, and the collision energy was 30 eV for IDA analysis. The resulting mass spectra were processed further using Progenesis QI Software (Non-linear Dynamics, Durham, NC, USA).

### Chromatographic conditions

The sample was separated using a Waters ACQUITY UPLC HSS T3 column (1.8 µm, 2.1 × 100 mm; Waters Corporation, Milford, MA, United States) with a UHPLC system (Shimadzu, Tokyo, Japan). The mobile phases consisted of water with 0.1% v/v formic acid A) and acetonitrile with 0.1% v/v formic acid B), and the column was maintained at 35°C. The separation was carried out using a linear gradient at a flow rate of 0.25 ml/min. Specifically, the gradient started at 2% B and increased to 60% B within 5 min, followed by a hold at 60% B for 5 min. Subsequently, the gradient was ramped up to 100% B for between 10 and 17 min and held at 100% B for 17–20 min. Finally, the gradient was decreased from 100% B to 2% B within 19–20.1 min. The sample volume injected was 5 µl.

### Data preprocessing

Progenesis QI software was used for LC-MS data analysis. The software performed peak picking, alignment, and area normalization using pooled QC injections as a reference. Features that were absent in less than 10% of the pooled QC injections were removed, and an Excel file was obtained with m/z, peak retention time (RT), peak intensities, and RT-m/z pairs as identifiers for each ion. Metabolites were identified using Progenesis QI data processing software, with the aid of public databases such as HMDB and LIPID MAPS, as well as in-house databases. The Progenesis QI score, fragmentation score, and isotope similarity were reported for all annotations based on accurate mass and fragmentation data. Metabolic pathways were analyzed using MetaboAnalyst 5.0 (Chong et al., 2018).

### Targeted metabolomics and measurement of serum biomarker levels

To prepare the samples, 20 µl of plasma was mixed with 80 µl of an internal standard consisting of d4-IAA and d5-Trp in methanol. The mixture was vortexed for 1 min at 4°C–8°C to precipitate proteins, and the supernatant was collected by centrifugation at 20,000 × *g* for 10 min at 4°C. For analysis, 1 µl of the supernatant was injected into the system, which used an analytical column (5 µm Kinetex EVO-C18 150 mm × 4.6 mm; Phenomenex, Torrance, CA, United States) with mobile phases A (0.1% formic acid in water, v/v) and B (0.1% formic acid in acetonitrile, v/v). The column was maintained at 35°C, and all analytes were detected in the positive ion multiple reaction monitoring mode. The transitions with m/z 205.1,146 were quantified for Trp, m/z 176.04,130.02 for IAA, m/z 190,144 for Kyna, and m/z 209,94 for Kyn using a scan time of 0.1 s per transition. Chromatographic separation of the analytes was achieved using a linear and fast gradient elution program consisting of a 0–0.5-min hold at 80%, 0.5–4.5-min decrease from 80% to 10% B, 1.5-min hold at 10% B, and finally an increase to 80%. The flow rate was maintained at 0.5 ml/min.

All MS parameters were optimized by direct infusion. The declustering potential and collision energies for specific quantification and confirmation transitions were optimized to maximize the sensitivity.

### Cell clustering and differential gene expression (DGE) analysis of scRNA-seq

Seurat is a popular R package used to analyze single-cell RNA sequencing data (Hao et al., 2021). In this study, Seurat v2.0.1 was used for quality control and filtering of the single-cell data. Highly variable genes were identified using the Find Variable Genes method of the Seurat package, and 2,000 genes were selected for further analysis, including principal component analysis (PCA). Principal components were used for cluster identification using the uniform manifold approximation and projection (UMAP) algorithm, which is a commonly used non-linear dimensionality reduction technique for visualizing high-dimensional data. Clusters were annotated to specific cell types based on the cell marker database. Finally, the FindMarkers function in Seurat was used to identify differentially expressed genes (DEGs) between healthy children and KD patients. All single-cell transcriptome data used in this study were obtained from a previous study (Shaw et al., 2021).

### Statistical analysis

All statistical analyses were performed using GraphPad Prism V.9 (GraphPad Software, San Diego, CA, United States) or SPSS version 25 (SPSS Inc., Chicago, IL, United States). Significance was assessed using one or more of the following: *t*-test, Mann–Whitney *U* test, Spearman’s rank correlation test, and chi-squared test. Receiver operating characteristic (ROC) curves analysis was performed using two-tailed unpaired Student’s *t*-tests (normal distribution) or Mann–Whitney U-tests (non-normal distribution). Comparisons between two groups for the remaining variables were performed using chi-squared or Fisher’s exact tests. Statistical significance was set at *p* < 0.05.

## Data Availability

The datasets presented in this study can be found in online repositories. The names of the repository/repositories and accession number(s) can be found below: https://www.ebi.ac.uk/metabolights/, MTBLS4983.
